# Whole grain intake, overall diet quality and key components of sustainable diets in Finnish adults

**DOI:** 10.1093/eurpub/ckac129.170

**Published:** 2022-10-25

**Authors:** R Tammi, S Männistö, H Reinivuo, H Tapanainen, J Rautanen, M Maukonen, E Päivärinta, A-M Pajari, NE Kaartinen

**Affiliations:** 1 Department of Public Health and Welfare, Finnish Institute for Health and Welfare, Helsinki, Finland; 2 Department of Food and Nutrition, University of Helsinki, Helsinki, Finland

## Abstract

**Background and objectives:**

Whole grains have been deemed a core component in diets promoting human health and environmental sustainability. Yet, research is scarce on whole grain intake in relation to overall diet quality and diet sustainability. We aimed to examine the association of whole grain intake with overall diet quality and key components of sustainable diets (fruits, vegetables, legumes, red and processed meat, plant-based and animal-based proteins) in Finnish adults.

**Methods:**

Our data comprised 3127 adults (58% women, energy underreporters excluded) aged 18 − 74 years participating in the population-based FinHealth 2017 Study. Dietary intake was assessed by a validated 134-item food frequency questionnaire. Food, nutrient, energy and whole grain intakes were calculated utilizing the Finnish Food Composition Database. Overall diet quality was examined by the modified Baltic Sea Diet Score (excluding cereals). Associations were assessed by linear regression analysis adjusted for relevant confounders.

**Results:**

Whole grain intake was positively associated with overall diet quality and fruit consumption (p < 0.001) in women and men. A positive association also occurred with plant-based protein intake (p < 0.001, women and men). Yet, whole grain intake was inversely associated with legume consumption in women (p = 0.001), while no association was found in men (p > 0.05). The association between whole grain intake and the intake of animal-based proteins and red and processed meat was inverse (p < 0.001) in both sexes. No association was found between whole grain and vegetable intakes (p > 0.05).

**Conclusions:**

Our results suggest that whole grain intake is associated with healthier diets and more sustainable protein intake in Finnish adults. However, challenges in furthering healthy and sustainable diets in the population may occur regarding legume consumption. Legumes are especially important in plant-based diets as they complement cereals as a source of essential amino acids.

**Key messages:**

• Higher whole grain intake may indicate higher overall diet quality and more sustainable protein intake in Finnish adults.

• Legume consumption requires further attention among Finnish adults with high whole grain intake despite their generally higher overall diet quality and more sustainable protein intake.

